# Being Pregnant during the Kivu Ebola Virus Outbreak in DR Congo: The rVSV-ZEBOV Vaccine and Its Accessibility by Mothers and Infants during Humanitarian Crises and in Conflict Areas

**DOI:** 10.3390/vaccines8010038

**Published:** 2020-01-22

**Authors:** David A. Schwartz

**Affiliations:** Medical College of Georgia, Augusta University, Augusta, GA 30912, USA; davidalanschwartz@gmail.com

**Keywords:** Ebola virus, Ebola vaccine, Democratic Republic of the Congo, pregnancy, maternal death, rVSV-ZEBOV, vaccination, hemorrhagic fever, excluding pregnant women, epidemic, filovirus, clinical trial, vaccinating pregnant women, humanitarian crisis, clinical trials, conflict area

## Abstract

The Ebola virus disease (EVD) outbreak that began in Kivu province of the Democratic Republic of the Congo (DRC) in July 2018 is the second largest in history. It is also the largest and most deadly of the ten Ebola outbreaks to occur in DRC, the country where Ebola was first identified during the 1976 Yambuku outbreak. The Kivu region is one of the most challenging locations in which to organize humanitarian assistance. It is an active conflict zone in which numerous armed groups are conducting violent acts, often directed against the inhabitants, healthcare and relief workers and peacekeepers. EVD has been especially problematic in pregnancy—previous outbreaks both in DRC and other countries have resulted in very high mortality rates among pregnant women and especially their infants, with maternal mortality in some outbreaks reaching over 90% and perinatal mortality 100%. The development and implementation of the Merck rVSV-ZEBOV vaccine for Ebola infection has been a tremendous public health advance in preventing EVD, being used successfully in both the West Africa Ebola epidemic and the Équateur DRC Ebola outbreak. But from the start of the Kivu outbreak, policy decisions had resulted in excluding pregnant and lactating women and their infants from receiving it during extensive ring vaccination efforts. In June 2019, this policy was reversed, 10 months after the start of the outbreak. Pregnant and lactating women are now permitted not only the rVSV-ZEBOV vaccine in the continuing Kivu outbreak but also the newly implemented Ad26.ZEBOV/MVA-BN vaccine.

## 1. Introduction

The Ebola virus disease (EVD) outbreak that began in the Kivu province of the Democratic Republic of the Congo (DRC) in 2018 is now the second largest in history [1]. It is also the largest and most deadly of the ten Ebola outbreaks to occur in DRC [2], the country where Ebola was first identified during the 1976 Yambuku outbreak [3]. Throughout multiple outbreaks of EVD, the infection has had a significant case fatality rate (CFR) among all persons who became infected, but historically the mortality has been especially high among pregnant women, fetuses and infants (less than 1 year of age) [4,5,6,7]. Prior to the West African Ebola epidemic which began in Guinea in December 2013, it was generally believed that EVD occurring in a pregnant woman was close to being non-survivable. Previous Ebola outbreaks had demonstrated that from 75 to greater than 90 percent of infected pregnant women had died as a result of EVD [4,6,8,9].

This high mortality rate is typical among filoviruses, a family of viruses to which the Ebola virus belongs. A related group of African filoviruses, the Marburg and Ravn viruses, also exhibit high mortality rates, and especially among pregnant women and their infants [8]. Until recently, there were no specific forms of treatment or prevention of Ebola virus infection—patients were treated symptomatically. However, this situation changed with the advent of the West Africa Ebola epidemic, which was to become the largest in history. By this time, there were not only several medications that had become available for testing, but a recombinant vaccine, the rVSV-ZEBOV (also termed V920) vaccine, produced by Merck that underwent its first human clinical trial during the epidemic.

This article reviews the history of Ebola virus outbreaks in DRC with particular attention to its effect on pregnant women and their infants, and examines the development, accessibility and distribution of the rVSV-ZEBOV vaccine to pregnant and lactating women during the ongoing outbreak that began in Kivu province in 2018. Also examined is the policy of exclusion of pregnant and lactating women and their infants from the clinical trials, and subsequently the distribution, of this vaccine through the most recent three Ebola epidemics [6,10,11,12,13,14,15,16,17,18,19,20,21,22].

## 2. Pregnancy and the Ebola Virus in Democratic Republic of the Congo

**Figure 1:**
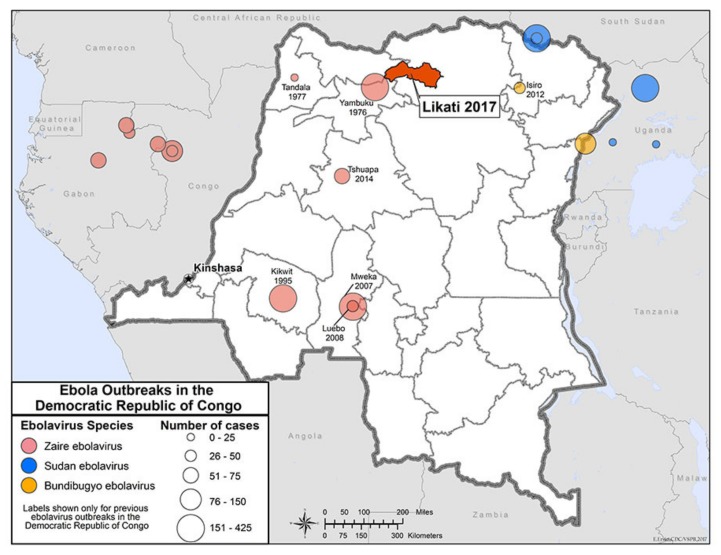
Map showing Ebola outbreaks in Democratic Republic of the Congo (formerly Zaire) up to 2017 and prior to the Bikoro, Équateur and Kivu outbreaks. Photograph from the US Centers for Disease Control and Prevention, Atlanta, USA.

Up to the present, there have been a total of 10 outbreaks of EVD in DRC. Those occurring prior to 2018 include outbreaks in Yambuku in 1976, Tandala in 1977, Kikwit in 1995, Mweka in 2007, Luebo in 2008, Isiro in 2012, Tshuapa in 2014, and Likati in 2017 (Figure 1). All outbreaks were caused by the Ebola Zaire subtype, except for the Isiro outbreak which was due to Ebola Bundibugyo. The outbreaks caused by the Ebola Zaire virus were between 51 and 75 cases; the Isiro outbreak involved 52 persons [23,24]. There has historically been a higher number of women infected compared with men—this is at least partly due to girls and women being the traditional caretakers of the sick and their significant involvement in funerary arrangements.

During outbreaks of Ebola virus and related viral infections in Africa, pregnant women face more severe consequences of disease, and have increased risks for morbidity, mortality and risk of fetal loss or congenital harm to the developing infant [9,25,26,27]. This is at least partially the result of such biological factors as the altered immune state in pregnancy, the role of the placenta in vertical transmission and immune modulation, and other physiological changes in pregnancy that can affect the clinical manifestations of disease [28,29,30]. In particular, women in the reproductive age group, and especially pregnant and lactating women, constitute a significant percentage of the female population in areas that viral hemorrhagic fevers strike. This is at least partly due to the high fertility rates and early ages of marriage in many regions where outbreaks occur. The DR Congo is the third most populous country in sub-Saharan Africa, has one of the highest fertility rates in the world, and is one of the few countries where the current fertility rate is greater than it had been in the 1950s [31]. Teenage pregnancy in DRC contributes to these metrics—27% of women age 15–19 have begun childbearing, while 21% are already mothers and 6% are currently pregnant. Adolescent fertility is nearly three times higher among young women living in the poorest households (42%) than among those living in the wealthiest households (15%) [32]. In the latest survey, the mean ideal number of children has been reported at 6.6, with even higher numbers in rural and poor areas [33]. Additionally, greater than one half (52%) of the household population in DRC is made up of children under age 15 [32]. As a result, women in the DRC spend a greater part of their lives pregnant and caring for infants than do women in other countries. In addition to these factors, the gender dynamics of girls’ and women’s caregiving roles, which include being the primary providers of care for the sick and involvement in funeral procedures, place them at heightened risks of exposure and greater rates of infection [34,35,36]. There can also be greater risks of Ebola virus exposure for pregnant women resulting from more frequent visits to healthcare settings for antenatal and obstetrical care, as occurred in the 1976 Ebola outbreak in Yambuku in which 46% of 177 women infected with Ebola were pregnant [9]. It is clear from these demographic data that withholding vaccine coverage to pregnant women and infants results in a great unmet need for protection during EVD outbreaks and ring vaccination programs.

While being pregnant and infected with Ebola virus is not new in DRC, having mothers and their infants survive the infection is. The case (maternal) fatality rate (CFR) among pregnant women with EVD during the initial outbreak in Yambuku was high—73 (89%) of the 82 infected women who were pregnant died, a case (maternal) mortality rate of 89% [6,37]. The rate of spontaneous abortions was 23% (19/82). During this initial outbreak in Yambuku, all 10 liveborn infants that were delivered to infected mothers subsequently died within 19 days of life from Ebola virus infection [6,13].

The highest maternal and infant CFRs reported for EVD occurred during the 1995 outbreak in Kikwit, DRC (then Zaire). Fifteen of the 105 (14%) Ebola-infected women were pregnant, and only one of the 15 EVD-infected pregnant women survived—a CFR of 93% [5,6]. Four pregnant women died from Ebola infection during the third trimester. The sole surviving mother among these women had a curettage because of an incomplete abortion after 8 months of amenorrhea [38]. However, it should be considered that all of these women were probably infected by means of the repeated use of contaminated needles for vitamin injections in routine antenatal care without sterilization between patients, and that this parenteral route of transmission is hypothesized to have contributed to the high CFR [6,39]. Perinatal infant mortality during the Kikwit outbreak was also high—10 women (66 percent) had spontaneous abortions, and one woman delivered a premature stillborn infant.

Following the Kikwit outbreak, an EVD outbreak occurred in Isiro, DRC in 2012. A 29-year-old mother at 7 months gestation developed EVD and had a premature delivery on day 6 of her infection; the mother succumbed to EVD one day following delivery, and the infant died at 8 days of life [23]. The Isiro outbreak eventually affected 58 persons, with 28 deaths.

Analysis of all EVD outbreaks in Africa prior to the West Africa epidemic reveals that there were 111 reported cases of pregnant women who had acquired the infection, with an aggregate CFR of 86% [9]. Every Ebola-infected pregnant woman that had survived did so only after a spontaneous miscarriage, elective abortion, stillbirth, or with a neonatal death [6]. All Ebola-infected pregnant women sustained vaginal and uterine bleeding and were at a significantly increased risk for both spontaneous abortion and pregnancy-related hemorrhage [6,40].

At the start of the West Africa Ebola epidemic in 2014, and based upon prior outbreaks in which close to 90 percent of infected pregnant women and 100 percent of fetuses died as a result of EVD, it was generally believed that almost all, if not all, pregnant women with Ebola infection would soon succumb to the disease [5,8,11,41]. In an interview performed early in the West Africa epidemic, a representative from a non-governmental organization had opined that the survival rate for expectant mothers was virtually zero [42]. In a report published in 2015 [43], the probability for maternal and infant survival of EVD was summarized as follows:

“*Present data suggests that maternal mortality remains high (approximately 95%) and peri-natal mortality virtually 100% for infected pregnant women*.”

In the beginning of the West Africa Ebola epidemic, there existed no approved specific treatments or vaccines for the infection. Clinical interventions consisted of supportive care, including fluid and electrolyte management, analgesia, support of blood pressure, management of coagulopathy, treatment of secondary infections, and a case-by-case management of other complications [11,40]. Medical treatments that had been proposed had not undergone clinical trials in Ebola virus-infected populations or at all [6]. Experimental vaccines were in the very early stages of development, and just a few had entered Phase I safety and immunogenicity trials. As the epidemic progressed and the fatalities increased, numerous proposals for clinical trials were submitted to the WHO Research Ethics Review Committee (WHO-ERC), the committee that evaluated and approved proposed potential clinical investigations including new and amended protocols for experimental interventional (drug, vaccine) and observational studies [44]. From August 2014 to April 2016, WHO-ERC evaluated 24 new EVD-protocols and 22 amendments, all of which excluded pregnant and lactating women and infants under 1 year of age. The proposed clinical trials for two promising antiviral drugs—brincidofovir and favipiravir—also excluded women who were pregnant for a variety of reasons (potential embryotoxicity for brincidofover and lack of insurance coverage for favipiravir) [13]. The WHO-ERC recognized that excluding pregnant women and their infants from drug and vaccine clinical trials undermined ethical principles of justice—fairness, equity and maximization of benefit, and that it would deny them the potential life-saving benefits from what was believed to be an almost universally fatal infection [13]. Because of this, the WHO-ERC systematically requested amendments to these protocols in order to include pregnant women and children. Despite their efforts as well as those of the Médecins Sans Frontières (MSF) Ethics Review Board and Inserm Institutional Review Board to have the applicants reconsider their exclusion of pregnant women, the necessity for rapid implementation of field trials had priority over the delays that would have been encountered in pursuing revision of the protocols to include pregnant women. The West Africa Ebola epidemic included many “firsts” for an Ebola epidemic—it was also the first time that a vaccine was fielded for preventative use for this infection. The rVSV-ZEBOV vaccine had been developed by the Canadian National Microbiology Laboratory, licensed to NewLink Genetics, and subsequently sublicensed to Merck, which has been the manufacturer and partner in ongoing research, licensure, and compassionate use efforts [39,45]. rVSV-ZW+EBOV is a live-attenuated vector vaccine using recombinant vesicular stomatitis virus to encode the glycoprotein of the Ebola Zaire strain. During its clinical testing conducted during the epidemic, an open-label, cluster-randomized ring vaccination trial (Ebola ça suffit!) of rVSV-ZEBOV in Guinea and Sierra Leone showed protective effects in non-pregnant adults. The WHO-ERC and Data Safety Monitoring Board requested that pregnant women receive the vaccine. Despite this, 42 pregnant women were denied participation in the trial [6,11]. During the West Africa epidemic, the rVSV-ZEBOV vaccine underwent extensive clinical study including multiple Phase I–III human clinical trials in non-pregnant persons to evaluate the safety and efficacy of the vaccine. But by the close of the epidemic, pregnant women, their fetuses and newborns had been systematically excluded from all vaccine clinical trials [5].

The first evidence indicative of the safety of administration of the rVSV-ZEBOV vaccine during pregnancy arose during the Ebola Phase III cluster-randomized ring vaccination trial in Guinea (Ebola ça suffit!) [6,13]. Because pregnancy tests were not being routinely performed, and a woman’s pregnancy status was based upon her own self-reporting, there were 23 pregnant women who were inadvertently administered the rVSV-ZEBOV vaccine [6]. Although the small sample size is suboptimal for statistical analysis and there was some loss of clinical follow-up, preliminary evaluation indicated that these women did not have significant differences in pregnancy loss when compared with non-pregnant women who received the vaccine [46]. Further preliminary data on the effects of the rVSV-ZEBOV vaccine among pregnant women have come from the Sierra Leone Trial to Introduce a Vaccine against Ebola (STRIVE), a randomized, unblinded Phase II/III trial conducted in 2015. Despite the exclusion criteria for this study including current pregnancy (pregnancy testing was required for all women <50 years old) as well as breastfeeding, there were 104 pregnancies that developed among 103 women (43 vaccinated, 60 unvaccinated) with an estimated onset within 2 months following vaccination or enrollment. Preliminary outcomes data among those women with known pregnancy outcomes have revealed no significant differences between vaccinated and non-vaccinated mothers [47].

While the West Africa Ebola epidemic was increasing in severity, on 24 August 2014 district officials in the DRC notified the WHO of an Ebola outbreak occurring in the Boende district in Équateur province. The index case of this outbreak was a pregnant woman—the wife of a bushmeat hunter. This pregnancy had a bad outcome—the mother died together with her fetus, who remained in utero [48]. Although this outbreak was at first believed to have a connection with the West Africa Ebola epidemic which was occurring at the same time, results of virologic analysis showed that the Ebola strain in the Boende outbreak was related to the Ebola strain that caused the Kikwit, Zaire (DRC) outbreak in 1995, and not the same strain as was spreading in West Africa [49].

Almost four years after the close of the West Africa epidemic, on 3 May 2018 health authorities from the Équateur province of northwestern Democratic Republic of the Congo reported that 17 persons had become ill with EVD near Bikoro, a small market town next to Lake Tumba south of Mbdanka, near the neighboring Republic of the Congo. This Équateur outbreak of Ebola virus was the ninth to occur in the DRC since the virus was first identified in 1976 [2]. The index case was identified to be a police officer. Following his funeral, 11 members of his family also developed the infection, of whom seven had provided care for him or attended his funeral. On 17 May, the first case of EVD was reported from Mbadanka, the capital city of Équateur province and a busy port city with over one million persons located on the Congo River. This was the first time that Ebola virus had reached a city in the DRC, and it rekindled fears of when Ebola virus had reached urban areas during the West African epidemic. In addition, there was concern that Ebola virus could spread via river traffic to the capital city of Kinshasa, a city of approximately 11 million, as well as to Brazzaville, both of which lie on the Congo River, and then across national borders to nine other countries as well [5,11]. Fortunately, rVSV-ZEBOV was by that time available for distribution. Clinical investigations had been initiated during the West Africa Ebola epidemic including the Partnership for Research on Ebola Vaccines in Liberia (PREVAIL; NCT02344407), Sierra Leone Trial to Introduce a Vaccine Against Ebola (STRIVE; NCT02378753), and the “Ebola ça suffit!” cluster-randomized ring vaccination trial in Guinea (PACTR201503001057193), which all strongly supported the protective effects of the single dose vaccination strategy with rVSV-ZEBOV [48,49,50], currently estimated to have a vaccine efficacy of 97.5% [51]. This was the first time that this Ebola vaccine, donated by Merck, was distributed early in the course of an EVD outbreak. Ring vaccinations were quickly organized in the affected areas—using this method, contacts of those infected, followed by contacts of those contacts, were vaccinated, as were healthcare workers, laboratory personnel, surveillance workers and people involved with burials. Unfortunately, the exclusionary policies that denied vaccine administration to pregnant and lactating women and infants that had been maintained during the West Africa epidemic were once again implemented, preventing them from receiving the potentially life-saving vaccine [11]. By the close of the epidemic on 24 July 2018, there were 54 confirmed or suspected cases and 33 deaths, with a CFR of 61%. A total of 3330 persons received the rVSV-ZEBOV vaccine during the outbreak [52]—none of them pregnant or under one year of age.

## 3. Conflict in Kivu

In order to understand the challenges of controlling an epidemic in the Kivu region of DRC, it is necessary to understand the nature of the hostilities that have been ongoing for over 25 years. This armed conflict is the world’s deadliest conflict since World War II, with over 5.4 million people dead and greater than 3 million displaced since the violence began in the mid-1990s. Following the multiethnic and multinational First Congo War (1996–1997) and Second Congo War (1998–2003), a rebel group supported by Rwanda sought the overthrow of the Congolese president Laurent-Désiré Kabila. A Congolese-borne Tutsi officer in the Rwandan-supported rebel group Rally for Congolese Democracy (*Rassemblement Congolais pour la Démocratie or* RCD), A rebel leader, Laurent-Desire Kabila, who was originally backed by Kagame, turned against him and kicked out the Rwandan forces, starting the second Congo war.several thousand Rwandan Hutus came into Kivu due to a political upheaval after the genocide of Tutsis and Hutu moderates by hardline Hutus several thousand Rwandan Hutus came into Kivu due to a political upheaval after the genocide of Tutsis and Hutu moderates by hardline HutusLaurent Nkunda, joined the new integrated national army of the DRC. After his promotion to General in 2004 Nkunda rejected the authority of the DRC government, retreating with RCD-Goma troops to the Masisi forests in North Kivu. Several thousand Rwandan Hutus had fled to Kivu due to a political upheaval after the genocide of Tutsis and Hutu moderates by hardline Hutus. Together with his troops, Nkunda conducted armed rebellion against the government of Joseph Kabila, thus initiating the Kivu Conflict. Nkunda claimed that his actions were to prevent genocide against the Banyamulenge, an ethnic Tutsi people residing in the South Kivu province of eastern DRC. However, many sources consider that the active and illegal trade in minerals and the financial gain in selling these resources to both national and international brokers has fueled this conflict, leading to the term for such minerals as “the engines of chaos” [53]. According to Jewish World Watch [54]:

“*Violence is being fueled by a multi-million dollar illicit mining industry of minerals such as the 3 Ts (tin, tantalum, and tungsten) that can be found in all of our electronic devices including: smartphones, gaming systems, computers, and military equipment*”

(“conflict minerals”).

Over the following years, armed conflict occurred primarily in North Kivu, but also extended into Katanga province and Burundi, involving multiple belligerent parties including the military of the Democratic Republic of the Congo (FARDC), the Hutu Power group Democratic Forces for the Liberation of Rwanda (FDLR), *Mouvement du 23 Mars* (M23), National Congress for the Defence of the People (CNDP), Mai Mai self-defence militias, Army for the Liberation of Rwanda (ALiR), Alliance of Patriots for a Free and Sovereign Congo (APCLS), Resisting Congolese Patriots (PARECO), and Allied Democratic Forces (ADF), to name just a few. In addition to these groups, smaller cohorts of armed individuals, collectively termed Mai Mai, are involved and led by warlords, traditional tribal elders, village heads, and politically motivated pro- or anti-government fighters. Since the Kivu conflict began, the effects of constant armed threats and conflict have resulted in the North and South Kivu provinces regressing into regions controlled by armed militia groups. It has been estimated by some researchers that there are currently greater than 70 armed groups operating in the Kivu region [55]; the Congo Research Group of New York University has estimated as many as 134 armed groups are operating in North and South Kivu [56]. Data from the Kivu Security Tracker, a joint project between Human Rights Watch and the Congo Research Group at New York University, indicates that between 1 June 2017 and 26 June 2019, there have been 3015 documented incidents of fighting and abuses in the Kivu provinces, involving 6555 victims [57]. These include violent deaths—1041 incidents with 1897 victims; mass rapes—24 incidents with 100 victims; abductions and kidnappings for ransom—848 incidents with 3316 victims; destruction of properties—148 incidents; political repression—106 incidents; and clashes—1290 incidents. These data, considered together with the most reliable available population statistics, are indicative of 8.38 civilians being killed per 100,000 people in the Kivu provinces for the single year 2018. Thus, the Kivu region is one of the worst imaginable places for an epidemic of Ebola virus to occur, as it continues to see levels of violence as high as some of the most violent places in the world—higher even than in Nigeria’s Borno State (the state most affected by Boko Haram and al-Qaeda in West Africa) and Yemen [57]. On 7 December 2017, armed rebels killed 14 United Nations peacekeepers in North Kivu province, targeting troops from Tanzania [58], making it one of the worst attacks on UN forces in recent history.

Some of the armed groups that operate in Kivu generate income from the export of coltan and cassiterite across the Goma/Gisenyi border and controlling the routes in and out of the regions; levying taxes on households and transport; profits from forest resources such as charcoal, timber and hemp; the pillaging of livestock; and organized theft [55]. Throughout the Kivu conflict, such gender-based atrocities as mass rape of girls and women and other forms of sexual violence have been employed by some belligerent groups as a weapon of war to instill fear in the broken communities. Although the precise number of rape victims cannot be determined, and is therefore unknown, a study performed in 2011 found that approximately 1152 women were raped every day, or in other words, 48 women raped an hour [59,60]. Other mass atrocities have included attacking villages, forced labor and the forced recruitment of children [56,61]. An MSF health worker in Nyabiondo, Anastasia Icyizanye, stated that one armed contingent of fighters raped 60 women after seizing a village market [62]. She said:

“*Whenever there is fighting there is systematic rape—in villages, at checkpoints on roads, wherever*.”

The atrocities against children are especially harmful and include boys as young as 14 being forced to fight on the battlefield under a spell of “*Juju*”, or black magic, so that they will not fear bullets [61]. Young girls described being abducted by armed groups and forced into marriage with their captors. A 2018 UNICEF report stated that the number of children recruited by armed groups as combatants and sexual slaves in the DRC is “significant” and worrisome [63]. Of particular significance to infectious disease epidemics is that these violent attacks, kidnappings and harassment have targeted clinics, hospitals, healthcare staff, vaccination team members, non-governmental agency personnel, and other relief workers attempting to provide care in the conflict areas [64,65,66,67,68]. This violence has continued throughout the Kivu Ebola outbreak—on 26 November 2019 the WHO and UNICEF evacuated 76 staff members from Ebola teams in the town of Beni due to a surge in violence, and WHO spokesman Christian Lindmeier stated [69]:

“*The violence has to stop, we have enough areas where we cannot go due to violence, military violence or rebel violence going on. So this is very bad for the Ebola response*.”

In reference to the violence, including attacks by rebel organizations including the Allied Democratic Forces (ADF) that are operating in the regions of Ebola virus relief and who attacked the Beni office of the United Nations Organization Stabilization Mission (MONUSCO) [70], WHO Director-General Tedros Adhanom Ghebreyesus, PhD, said:

“*This could prolong the outbreak. I’m concerned about the well-being of responders & communities. I call on all parties to halt the violence*.”

The sad truth is that all parties involved in this conflict—the official army of the DRC and those of neighboring governments, armed rebel groups, and the United Nations peacekeepers—have committed atrocities against the indigenous populations [60,71]. According to Michael VanRooyen, the director of the Harvard Humanitarian Initiative [59]:

“*…the important message remains: that rape and sexual slavery have become amazingly commonplace in this region of the DRC and have defined this conflict as a war against women*.”

Unfortunately these violent acts against villages, rape of women and girls, attacks against healthcare and relief workers, forced labor and the recruitment of children continue to be committed to this day.

## 4. The Kivu Ebola Outbreak

Just one week following the close of the Équateur province outbreak, Ebola returned to the DRC for the tenth time—this time to the northeastern region of the country [72,73]. In the town of Mangina in North Kivu district, a woman presented to a local health center on 19 July 2018 for a cardiac condition. She subsequently died at home with symptoms of hemorrhagic fever on 25 July. Following her death, several members of her family developed the same symptoms and died shortly afterwards [5,74]. A team of investigators identified an additional six cases of the same illness, which was confirmed to be due to the Ebola virus, and an outbreak was officially declared on 1 August 2018.

This new outbreak developed in a region of DRC that was one of the most challenging locations to conduct epidemiologic surveillance and implement public health, social support and medical intervention. North Kivu province is a densely populated region that borders Uganda to the east and Rwanda to the south. As summarized above, the North Kivu region of DRC had been embroiled in a series of civil wars and armed conflicts for decades. There were more than 100 armed groups operating in this region [74], in which there were intensive military operations ongoing. The administrative center of the district, Beni, was under military rule. The presence of “red zones”—areas that are inaccessible to public health workers due to fighting and the risk of kidnapping—prevented health workers from working where surveillance and treatment were needed. This was the first EVD outbreak occurring in a war zone, where armed rebel militias constituted an ever-present threat to healthcare and public health workers, physicians and nurses, epidemic control personnel and other members of the humanitarian relief staff.

Based on the success of the ring vaccination strategy during the West Africa epidemic and Équateur outbreaks, the decision was made to use the same approach in Kivu. Vaccination using the rVSV-ZEBOV vaccine was begun on 8 August, and a ring vaccination program was implemented in an attempt to stop spread of transmission. In this program, the rVSV-ZEBOV vaccine was offered to contacts of known cases and the contacts of contacts, including any individual over 1 year of age. The DRC Ministry of Health, together with WHO and other partners, decided that pregnant and lactating women would, once again, be excluded from receiving rVSV-ZEBOV, the only Ebola vaccine to have completed efficacy testing. Thus, for the third sequential Ebola epidemic/outbreak, the rVSV-ZEBOV was administered to persons at risk but excluded pregnant and lactating women and infants [1,11,15].

Within the public health community, this policy of withholding vaccination was considered by many to be indefensible, especially given that there had never been a mother–infant pair that had survived EVD [10,11,21]. Three public health experts from Johns Hopkins University [10] wrote:

“*The rVSV-ZEBOV vaccine will give pregnant women, and the children they are carrying, a chance to live. Without it, most of the pregnant women infected with Ebola, and almost all of their infants, will die*.”

WHO’s Strategic Advisory Group of Experts (SAGE) on Immunization met in October 2018 in Geneva to discuss the development of candidate Ebola vaccines and the progress made in implementing the “expanded access and compassionate use” vaccine protocol in the DRC, but no recommendation for immunizing pregnant women was made. At this meeting, SAGE reviewed the available data for rVSV-ZEBOV based upon clinical outcomes from those women who had been inadvertently vaccinated in early pregnancy or women who became pregnant shortly after being immunized [75,76]. SAGE conceded that significant data gaps existed, and that there was loss to follow-up and a lack of an appropriate control group in most instances. Within these constraints, the investigators did not detect any statistically significant increase in the risk of pregnancy loss associated with periconception or perinatal vaccination, but neither could they exclude it. Given the gaps in data, SAGE did not believe that enough information existed at the time to issue a definitive recommendation on whether pregnant women should be offered the vaccine, and thus deferred to local authorities in the DRC to determine the ongoing strategy [39]. They stated:

“*Additional safety data among other target populations such as children, HIV-positive individuals and pregnant women is required*.”

The Ebola outbreak continued to rapidly spread, and by November 2018 the infection was reported from two provinces—North Kivu and Ituri—as well as 14 health zones. Surveillance data revealed that there was a predominance of women who were becoming infected, especially in the reproductive age group [10]. Women of child-bearing age were not the only persons that were significantly affected—infants and children made up greater than one third of all Ebola infections [77], with one in 10 cases of EVD occurring in children less than 5 years of age. As of 12 January 2019, a total of 595 confirmed and 49 probable cases of EVD had occurred in 16 health zones in DRC, with a CFR of 58% among confirmed cases—a total of 59,453 persons had received the rVSV-ZEBOV vaccine, none of them pregnant or less than one year of age [78]. Social science data analysis demonstrated that the community members had heard of the existence of the vaccine, were generally in favor of the use of vaccines to prevent disease and were widely receptive to it [79,80]. However, there existed confusion about the inclusion/exclusion criteria for vaccination, with community members asking: ‘*why are some vaccinated and some not?*’ Qualitative data collected by UNICEF from pregnant and lactating women in Beni in October 2018 demonstrated their concern that children (who they regarded to be at high risk because ‘*they play in decontaminated houses*’ and ‘*sleep in the beds of their mothers*’) were not being routinely vaccinated. In Beni and Butembo, only 66% of respondents agreed with the statement that they ‘*believe the Ebola vaccine works*’, and just 63% stated that they would accept the vaccine if it was available to them. Among the 37% of interviewees who had said they would refuse the vaccine, 71% believed it was ‘*dangerous*’ and 23% believed that it did not work (in comparison to 12% of the total survey respondents who thought vaccines, in general, were unsafe and 9% that vaccines did not work) [79,80].

In addition to calls for the inclusion of pregnant women and their infants in vaccine administration from the public health, biomedical and bioethics communities, pregnant women and other community members from the DRC who were directly affected by the epidemic began to make their voices heard, clearly stating that pregnant women should be able to make the decision for themselves whether or not to receive the vaccine [79,80]. In interviews [39,79] conducted with pregnant women in the city of Beni, there were comments from pregnant women regarding their exclusion from vaccination such as:

“*Now there is no option, you just send us to death*.”

“*You tell us to protect yourself with the vaccine, and then you tell us we cannot get the vaccine. So we have nothing left*.”

“*You told us to accept [the vaccine] and now we do, but now you don’t give it to save us*.”

As the Ebola epidemic expanded during the beginning of 2019, there were additional calls to reassess the exclusionary policy of withholding the vaccine from pregnant and lactating women and their infants. By the close of January 2019, the DRC National Institute for Biomedical Research had begun revising the vaccination protocol to include pregnant and lactating women within Ebola contact rings in their ongoing immunization efforts. This new policy was endorsed and supported by WHO SAGE at their meeting in Beijing on 20 February 2019. SAGE had made the decision to support pregnant and breastfeeding women and infants receiving the Ebola vaccine [81], noting that:

“*In view of the severity of the outbreak and aligned with SAGE’s recommendation from October 2018 [1], SAGE welcomes and supports the recent recommendation of the ethics committee of DRC to also authorize the vaccination of pregnant women in outbreak affected areas, using the currently recommended vaccination strategies, with the live-replicating rVSV-ZEBOV-GP vaccine with informed consent and in compliance with GCP. As recommended by the ethics committee, every effort must be made to collect data on the safety of the vaccine in these populations, including a documentation of the pregnancy outcomes. SAGE advises that the use of rVSV-ZEBOV-GP vaccine in pregnant women currently remains limited to the EVD outbreak affected areas in DRC and should be continuously evaluated based on the emerging data on the safety and efficacy of the vaccine in this target population*.”

By the time this policy change was announced, there had already been 773 confirmed cases, 65 probable cases, and 534 deaths; 57% of the confirmed and probable EVD cases were female, and 61% of them were of child-bearing age [81,82,83]. The policy reversal was met with great approval from the public health community [14,84]—it was anticipated that it would provide an example for the inclusion of pregnant women in the design and clinical trials of vaccines that were in development for such infections as Zika virus and Lassa virus [11,39,85].

Despite this landmark decision by SAGE and the continued spread of the Ebola outbreak, pregnant and lactating women and their infants were still not permitted to receive the rVSV-ZEBOV vaccine [11,12]. From 26 November 2018 to 26 May 2019, there were 319 pregnant women and 603 lactating women registered as Ebola contacts, but all were denied the vaccine and were at risk for acquiring the infection [11,12]. In the case of Josephine Kakule, an unvaccinated 26-year-old pregnant woman who acquired EVD in her eighth month of pregnancy in early 2019, the future looked grim. She lived in a village located 100 m from Kivu—an active conflict zone—and following her diagnosis and isolation in a treatment center in Kivu, she said:

“*It was painful to separate from my family and my children but I had to do it*.”

Josephine was one of the lucky ones—she survived the infection, and two weeks after being declared Ebola-free went on to deliver an uninfected baby girl [86].

**Figure 2:**
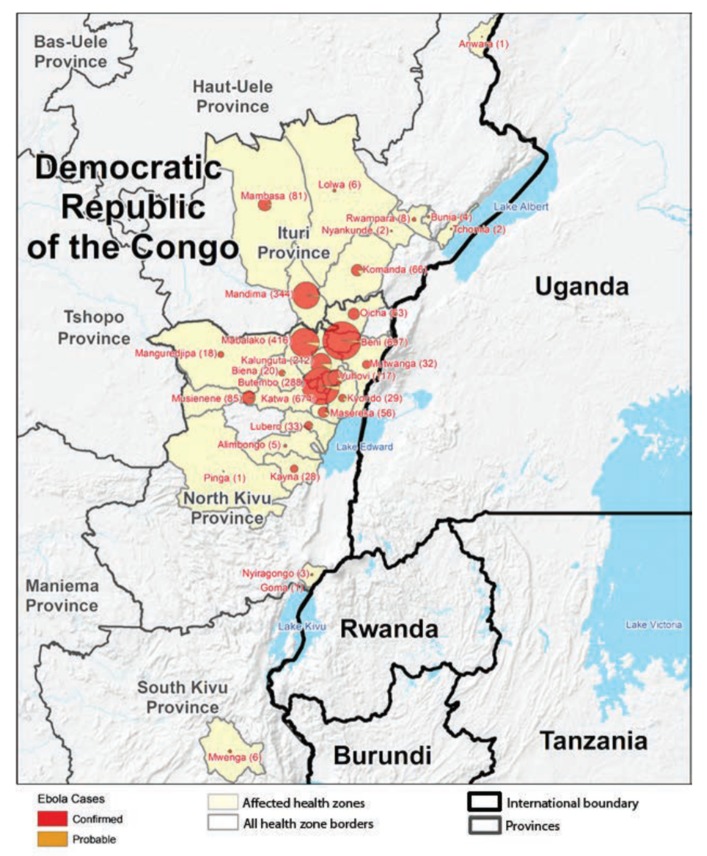
Map of the Kivu Ebola outbreak. Geographic distribution of confirmed and probable cases of Ebola virus disease (Ebola) by health zones—North Kivu, South Kivu, and Ituri provinces, Democratic Republic of the Congo, 30 April 2018–17 November 2019. From the US Centers for Disease Control and Prevention, Atlanta, USA.

By June 2019, the Kivu Ebola outbreak had resulted in 2000 cases and 1339 deaths—it was the second largest outbreak of Ebola virus infection in history (Figure 2). The rapidity of spread and transmission dynamics of the epidemic were evidenced by the fact that although it took 8 months to reach one thousand infected persons, in just 71 days another thousand individuals developed the infection [87]. This rapid increase in new EVD cases and doubling of the total number of infected persons corresponded closely in time with a surge in acts of violence against healthcare operations as well as directed against the civil population. On 19 April, there was an attack against a hospital in Katwa by armed militia members that resulted in the death of Dr. Richard Mouzoko Kiboung, a WHO epidemiologist, and injury of two other healthcare workers [88]. In the same month, it was estimated that up to 60,000 persons fled their homes as a result of fighting around Kamango near the town of Beni, and an additional 50,000 fled in the neighboring Lubero Territory due to violence between the Congolese Army and Mai Mai armed groups [89].

The cause of delay in initiating vaccination of pregnant women and infants was the result of modifications to the immunization protocol requested by the DRC’s National Ethics Committee [15]. This months-long delay in vaccinating put hundreds of pregnant women and their fetuses at risk. According to Professor Steve Ahuka, Director of Virology at DRC’s National Institute of Biomedical Research, these modifications included women in their first trimester of pregnancy being excluded from vaccination. Dr. Ahuka said [15]:

“*The vaccination of pregnant women is usually a very complex decision to make, especially when they are using a new vaccine which is still under a trial*.”

Finally, on 2 June 2019—almost four months after the process to revise the protocol to include pregnant women was initiated by SAGE—the DRC Ministry of Health announced that the National Ethics Committee at the School of Public Health at the University of Kinshasa had granted their approval to an amended vaccination protocol that would allow the administration of the Merck rVSV-ZEBOV vaccine to pregnant women beyond their first trimester of pregnancy and to lactating women if they were identified as case contacts. The committee continued to maintain that only children over the age of 6 should be vaccinated [12,15]. It had taken over 4 years for pregnant and breastfeeding women to receive EVD vaccination since the initial clinical trials [11]. The first pregnant women finally received the rVSV-ZEBOV vaccine on 13 June—almost 10 months after the outbreak began. According to Dr. Carleigh Krubiner, a Policy Fellow at the Center for Global Development [15]:

“*Pregnant and lactating women in the DRC finally have access to … one of the best prevention tools we have against this deadly virus … Hopefully this will set a new precedent for ongoing and future Ebola vaccination efforts, avoiding costly delays in protocol approvals while women face the very real threats of Ebola infection*.”

As of early October 2019, over 840 pregnant women in the Ebola outbreak zone of DRC have received the rVSV-ZEBOV vaccine [39]. At this early phase in the administration of the vaccine to pregnant women and their infants, data regarding maternal, fetal, or neonatal indicators and outcomes are still being collected.

## 5. The Ad26.ZEBOV/MVA-BN Vaccine Becomes Available Including Pregnant Women and Children

On 7 May 2019, the Strategic Advisory Group of Experts (SAGE) issued new recommendations that included introducing an additional experimental Ebola vaccine developed by the Janssen Division of Johnson & Johnson [90]. This vaccine, an adenovirus 26 vectored glycoprotein/MVA-BN-Filo (Ad26.ZEBOV/MVA-BN) vaccine regimen, had been designated as the second vaccine to be used in the outbreak. The two-dose vaccination regimen includes Ad26.ZEBOV as the first dose, which is based on Janssen’s AdVac^®^ technology, and MVA-BN-Filo as the second dose, which is based on Bavarian Nordic’s MVA-BN^®^ technology and is administered approximately 8 weeks later. This vaccination regimen uses a viral vector strategy in which viruses—adenovirus serotype 26 (Ad26) and Modified Vaccinia Ankara (MVA)—have been genetically modified so that they cannot replicate in human cells, while safely inducing the production of Ebola virus proteins in order to trigger an immune response [91]. Because of the inability for viral replication to occur in vaccinated persons, it was believed that this regimen might provide an option that health officials would be more comfortable recommending for pregnant women.

On 12 November 2019, the DRC’s technical committee (CMRE) announced that vaccination would be initiated on 14 November 2019 in two health areas of Goma, the capital city of North Kivu province, using the newly developed second vaccine. Everyone would be eligible to receive this vaccine, including children over the age of one year, as well as pregnant and lactating women. Women of childbearing age will be offered a pregnancy test, and if declined, will still receive the J&J vaccine. Pregnant women who receive the Ad26.ZEBOV/MVA-BN vaccine will be followed, according to Dr. Véronique Urbaniak, the Vaccine Project Coordinator at MSF, France [92].

## 6. Conclusions

Pregnant and lactating women and their infants have been continually denied access to vaccination for Ebola virus disease since the development and clinical trials of the Merck rVSV-ZEBOV vaccine, beginning with the West Africa Ebola epidemic in 2014. In spite of calls from the public health, biomedical and bioethics communities, this policy of exclusion continued through two subsequent Ebola virus disease outbreaks in DRC, including the Équateur province outbreak in 2018 and, just one week after its termination, the Kivu outbreak. The Kivu outbreak has been a humanitarian crisis that originated in an active conflict zone, which has severely affected the ability of relief efforts and vaccination, and has developed into the second largest Ebola outbreak in history. The previous exclusion of vaccination of pregnant women and infants continued into this epidemic, despite the fact that there was a predominance of women, children and infants who developed Ebola infection. By the beginning of June 2019, there were 131,000 persons, none of them pregnant, who had been administered the life-saving vaccine. Eventually, reevaluation of the restrictive policies resulted in the accessibility of pregnant and lactating women for vaccination, and ten months after the epidemic began the first pregnant women received the rVSV-ZEBOV vaccine.
